# Safety Assessment and Hypoglycemic Effect of *Moringa oleifera* Lam. Seed Lectin (WSMoL) in Alloxan‐Induced Diabetic Mice

**DOI:** 10.1002/jat.70161

**Published:** 2026-03-18

**Authors:** Alícia Natalie Silva dos Santos, Matheus Cavalcanti de Barros, Cleidiane Silva Vieira de Souza, Ryan Cristian da Silva, Luana Cassandra Breitenbach Barroso Coelho, Maria Tereza dos Santos Correia, Fernanda das Chagas Ângelo Mendes Tenório, Alisson Macário de Oliveira, Thiago Henrique Napoleão, Robson Raion de Vasconcelos Alves, Patrícia Maria Guedes Paiva

**Affiliations:** ^1^ Departamento de Bioquímica, Centro de Biociências Universidade Federal de Pernambuco Recife Brazil; ^2^ Departamento de Histologia e Embriologia, Centro de Biociências Universidade Federal de Pernambuco Recife Brazil

**Keywords:** diabetes mellitus, plant bioactive proteins, toxicological survey

## Abstract

*Moringa oleifera* Lam. seeds contain WSMoL, a lectin previously studied for toxicity and pharmacological effects. In this study, we investigated WSMoL (10, 25, or 50 mg/kg i.p.) for acute and 14‐day repeated‐dose toxicity, as well as genotoxicity, in Swiss mice. In addition, the hypoglycemic activity of WSMoL (25 and 50 mg/kg i.p.) was evaluated in alloxan‐treated mice. Animals were monitored for body weight, water and food consumption, behavioral changes, hematological and biochemical parameters, and histological patterns of the liver, spleen, and kidneys. Acute toxicity assay showed that WSMoL did not cause mortality or changes in behavior, food and water intake, or body weight. However, its administration resulted in monocytopenia, and histological changes consistent with an inflammatory process. The repeated‐dose assay also indicated no significant alterations in body weight, water, or food intake. Hematological analyses, however, revealed monocytopenia and lymphocytopenia, and histological changes consistent with inflammation were observed. The micronucleus test in bone marrow cells indicated that WSMoL was not genotoxic. Administration of WSMoL (25 and 50 mg/kg) significantly reduced fasting blood glucose levels and improved oral glucose tolerance in both male and female animals after 10 days of treatment. Interestingly, WSMoL at 50 mg/kg reduced blood glucose to levels comparable to insulin. Histological analyses suggest that WSMoL prevented liver and kidney damage induced by hyperglycemia. Overall, our results indicate that WSMoL is a promising natural alternative for diabetes treatment, but its use should be approached with caution due to potential subclinical toxicity.

## Abstract 2

This study evaluated the toxicity, genotoxicity, and hypoglycemic effect of the lectin WSMoL, isolated from *Moringa oleifera* seeds, in Swiss mice. WSMoL did not cause mortality or alterations in weight and food consumption, although it induced hematological and inflammatory changes. It did not show genotoxicity. In diabetic mice, it reduced blood glucose, improved glucose tolerance, and prevented liver and kidney damage. WSMoL is a promising natural alternative for diabetes treatment.

## 1 Introduction

Type 1 diabetes mellitus (T1DM), characterized by autoimmune destruction of insulin‐producing β‐cells in the pancreas, is a disorder that has been increasingly diagnosed in older adults and is associated with sequelae such as retinal and renal diseases, as well as an increased risk of cognitive decline, depression, and frailty (Tomic et al. 2025). Although insulin therapy is essential and effective for managing T1DM, researching natural products with hypoglycemic effects remains valuable since they may offer complementary benefits by improving blood sugar control, reducing the required insulin dose, and minimizing side effects. Additionally, they often possess anti‐inflammatory and antioxidant properties that could help prevent diabetes‐related complications (Usai et al. 2022; Uti et al. 2025).

Lectins are proteins that bind carbohydrates and exhibit multiple bioactivities, such as antitumor, antimicrobial, and antihyperglycemic activities (Patriota et al. 2019; Tsaneva and Van Damme 2020; Simha et al. 2024; Napoleão et al. 2025). The insulin receptor and GLUT transporters are glycoproteins whose glycosylation plays an important role in their function. Some lectins have been shown to recognize their glycan moieties and may even influence their activity (Hedo et al. 1981; Rouiller et al. 1986; Samih et al. 2003; Masnikosa et al. 2008; Grácio et al. 2021), making lectins particularly interesting for glucose regulation. A lectin from the inner bark of *Crataeva tapia* L. (CrataBL), when applied intraperitoneally for 10 days at doses of 10 and 20 mg/kg, showed hypoglycemic activity in alloxan‐induced diabetic mice and improved renal and hepatic alterations commonly associated with diabetes (Rocha et al. 2013). In turn, rats bearing streptozotocin‐induced diabetes showed a reduction in levels of glucose, cholesterol, total cholesterol, and triglycerides when treated with a lectin from *Bryothamnion seaforthii* (Turner) Kütz (BSL) at 150–600 μg/kg by gavage (Alves et al. 2020).

*Moringa oleifera* Lam. (Moringaceae) is a plant found in tropical and subtropical regions, commonly referred to as the “miracle tree” due to the medicinal and nutritional uses of its seeds, roots, leaves, and fruits (Pareek et al. 2023; Akabari et al. 2022). Its pharmacological properties include antioxidant, anti‐inflammatory, antidiabetic, anti‐ulcer, immunomodulatory, neuromodulatory, and wound‐healing effects, among others (Divya et al. 2024).

*M. oleifera* seeds contain lectins, including WSMoL (water‐soluble *M. oleifera* seed lectin), a 60 kDa anionic protein (pI 5.5) that appears as three polypeptide bands of 10, 20, and 30 kDa on sodium dodecyl sulfate–polyacrylamide gel electrophoresis (SDS‐PAGE), and whose hemagglutinating activity is inhibited by *N*‐acetylglucosamine, glucose, and fructose (Moura et al. 2016). WSMoL has been reported to have anti‐inflammatory (Araújo et al. 2013), antitumor (Brito et al. 2023), immunomodulatory (Coriolano et al. 2018), and psychotropic effects (Patriota et al. 2025). It also showed hypoglycemic effect in mice with Type 2 diabetes mellitus (T2DM), decreasing insulin resistance and improving cardiac function (Vera‐Nunez et al. 2021).

Safety evaluation is critical for the therapeutic use of plant‐derived compounds. While some lectins are beneficial, others can be highly toxic. For example, ricin, a lectin from *Ricinus communis* L. seeds, is lethal even at nanogram doses (Fu et al. 1996). Regarding WSMoL, Brito et al. (2023) reported that a single intraperitoneal dose of 100 mg/kg decreased food and water intake, reduced weight gain, and altered hematological, biochemical, and histological parameters in mice. A higher dose of 200 mg/kg caused 60% mortality.

Given previous reports on the toxicity of WSMoL, as well as its hypoglycemic effect in a T2DM model, this study aimed to expand knowledge on these aspects by assessing its acute and repeated‐dose (14‐day) toxicity, as well as its genotoxicity, following intraperitoneal administration, and to determine whether it also exhibits hypoglycemic efficacy in alloxan‐treated mice, thereby exploring its potential as a complementary treatment for T1DM.

## 2 Materials and Methods

### 2.1 Purification of WSMoL

*M. oleifera* seeds were collected in the city of Recife, Pernambuco, northeastern Brazil (8°02′58.3″ S 34°56′48.2″ W), with authorization of the *Instituto Chico Mendes de Conservação da Biodiversidade* (ICMBio, license 72024). One specimen (voucher no. 73,345) was deposited in the *Dárdano de Andrade Lima* Herbarium of the *Instituto Agronômico de Pernambuco* (Recife, Brazil).

WSMoL was purified following the methodology described by Coelho et al. (2009). Initially, 10 g of seed powder was homogenized in 100 mL of distilled water at 4°C for 16 h using a magnetic stirrer. The resulting mixture was centrifuged at 3000 × *g* for 15 min, and the supernatant was collected as the crude extract. Proteins in the extract were precipitated with ammonium sulfate at 0%–60% saturation (Green and Hughes 1955). The precipitated fraction was recovered by centrifugation, dialyzed against distilled water for 4 h, followed by dialysis in 0.15 M NaCl for an additional 4 h, and then applied to a chitin (Sigma‐Aldrich, St. Louis, MO, USA) column pre‐equilibrated with 0.15 M NaCl. WSMoL was eluted from the column using 1.0 M acetic acid and dialyzed in distilled water for 4 h. Protein concentration was quantified using the method of Lowry et al. (1951), with a bovine serum albumin (BSA; Sigma‐Aldrich) standard curve ranging from 31.25 to 500 μg/mL. Hemagglutinating activity was assessed in microtiter plates according to Procópio et al. (2017), employing a 2.5% (v/v) suspension of glutaraldehyde‐treated rabbit erythrocytes. One unit of hemagglutinating activity (titer^−1^) was defined as the reciprocal of the highest dilution of the sample that caused complete hemagglutination.

### 2.2 Animals

The protocols were approved by the Ethics Committee of *Universidade Federal de Pernambuco* (UFPE) (authorization nos. 0043/2022 and 130/2022). One hundred and 15 Swiss albino mice ( *Mus musculus* L.), comprising 65 females and 50 males, were obtained from the bioterium of the *Instituto Keizo Asami* (iLIKA) of the UFPE. Animals selected for the study were 6–8 weeks old and weighed between 30 and 35 g. Prior to the experiments, the mice were acclimated for 5 days in the animal facility of the *Departamento de Bioquímica* at UFPE, maintained at 21 ± 1°C under a 12:12 h light–dark cycle, with free access to food (Purina, São Paulo, Brazil) and water. The animals were randomly divided into groups using a simple randomization method. All mice were included in the final analysis, with no exclusions necessary. To minimize potential confounding factors, treatments were administered in the following order: negative control, WSMoL‐treated groups in ascending doses, and positive control (when applicable). Group allocation was only known to the researcher responsible for administering the treatments at each stage. No animal showed signs of toxicity corresponding to humane endpoints (pronounced weight loss, dehydration, loss of walking ability, inability to access food or water, and labored respiration) before the end of the test.

### 2.3 Acute and Repeated Dose Toxicity Assays

For the acute toxicity study, female mice were used according to the recommendations of OECD (2002) since they are generally slightly more sensitive. The animals were assigned to four groups (*n* = 5 per group) and treated with either 0.15 M NaCl (control) or WSMoL at doses of 10, 25, or 50 mg/kg administered intraperitoneally (i.p.). Dose selection was based on prior findings by Brito et al. (2023), who evaluated WSMoL toxicity at 100 and 200 mg/kg i.p. Each animal received a single treatment and was monitored for up to 2 h to detect mortality as well as the behavioral alterations outlined by Brito et al. (2023). Body weight, water consumption, and food intake were measured daily for 14 days. Fourteen days after administration, animals were euthanized with 300 mg/kg ketamine i.p. combined with 30 mg/kg xylazine i.p., and blood, as well as liver, kidney, and spleen samples, were collected for biochemical, hematological, and histopathological analyses.

In the repeated‐dose toxicity study, animals were assigned to eight groups (*n* = 5 per group, with separate male and female groups) and received either 0.15 M NaCl (control) or WSMoL at doses of 10, 25, or 50 mg/kg via intraperitoneal (i.p.) injection. Treatments were administered once daily for 14 consecutive days. Throughout the study, animals were monitored daily for mortality and behavioral changes (Brito et al. 2023), and their body weight, food intake, and water consumption were recorded. At the end of the treatment period, animals were euthanized with an intraperitoneal injection of 300 mg/kg ketamine and 30 mg/kg xylazine. Blood samples and organs (liver, kidney, and spleen) were collected for subsequent biochemical, hematological, and histopathological analyses.

### 2.4 Genotoxicity Assessment

Genotoxicity was evaluated using the micronucleus test in bone marrow cells. Female mice were assigned to groups (*n* = 5 per group) and treated intraperitoneally (i.p.) with 0.15 M NaCl (negative control), methotrexate at 25 mg/kg (positive control), or WSMoL at doses of 10, 25, or 50 mg/kg. After 24 h, animals were euthanized via i.p. injection of a ketamine (300 mg/kg) and xylazine (30 mg/kg) solution. The femurs were excised, dissected, and cut at the proximal epiphyses. Fetal bovine serum (1 mL per femur) was injected into the medullary cavity to flush out bone marrow contents, which were collected in tubes containing 2 mL of fetal bovine serum. The cell suspensions were centrifuged at 800 × g for 5 min, the supernatant (3 mL) was discarded, and the pellet resuspended in phosphate‐buffered saline (PBS) for slide preparation. Slides were air‐dried, fixed in methanol for 10 min, and stained with Leishman's methylene blue for 24 h (Schmid 1984). Micronuclei were assessed under a Nikon Eclipse E100 optical microscope, counting 1000 polychromatic erythrocytes (PCE) per animal to determine the frequency of micronucleated cells (MnPCE).

### 2.5 Hypoglycemic Activity and Oral Glucose Tolerance Test

Male and female mice were fasted for 12 h prior to the induction of diabetes. Alloxan monohydrate (Sigma‐Aldrich) was administered intraperitoneally (i.p.) at a dose of 180 mg/kg, dissolved in 0.15 M NaCl. Five days after injection, blood glucose levels were measured using a glucometer (OneTouch Ultra, Johnson & Johnson, New Brunswick, NY, USA). Animals exhibiting postprandial blood glucose concentrations greater than 250 mg/dL, along with characteristic diabetic symptoms such as polyuria, polydipsia, and polyphagia, were classified as hyperglycemic and selected for inclusion in the experimental protocol.

Animals were randomly assigned to five groups (*n* = 5 per group, with males and females analyzed separately): normoglycemic mice treated with 0.15 M NaCl (Sham), hyperglycemic mice treated with 0.15 M NaCl (negative control), hyperglycemic mice treated with insulin at 3 U/day (positive control), and hyperglycemic mice treated with WSMoL at the nonlethal doses of 25 or 50 mg/kg. All treatments were administered intraperitoneally (i.p.) for 10 consecutive days (Rocha et al. 2013). Throughout the experimental period, blood glucose, body weight, and water and food intake were monitored daily. On the tenth day of treatment, an oral glucose tolerance test (OGTT) was conducted for all experimental groups. Mice received an oral glucose load of 2.5 g/kg, and blood glucose levels were measured at baseline (*t* = 0) and subsequently at 30, 60, and 120 min following glucose administration (Jenkins et al. 2021). After completion of the test, animals were euthanized via intraperitoneal injection of ketamine (300 mg/kg) and xylazine (30 mg/kg). Blood, liver, and kidney samples were then collected for biochemical, hematological, and histopathological analyses.

### 2.6 Hematological and Biochemical Analysis

Blood samples were collected into tubes containing separating gel for biochemical assays and into tubes with ethylenediaminetetraacetic acid as an anticoagulant for hematological evaluations. Serum concentrations of total protein, albumin, alanine aminotransferase (ALT), aspartate aminotransferase (AST), alkaline phosphatase (ALP), gamma‐glutamyl transferase (GGT), urea nitrogen, and creatinine were determined using commercial diagnostic kits (Labtest Diagnóstica, Lagoa Santa, Brazil). Hematological parameters were analyzed with an automated hematology analyzer (Animal Blood Counter—ABC Vet, Montpellier, France) and confirmed by light microscopy. The following indices were assessed: erythrocyte count, hemoglobin concentration, hematocrit, mean corpuscular volume (MCV), mean corpuscular hemoglobin (MCH), mean corpuscular hemoglobin concentration (MCHC), and total and differential leukocyte counts.

### 2.7 Histological Analysis

Collected organs were sectioned and fixed in 10% (v/v) buffered formalin, followed by dehydration through graded ethanol solutions, clarification in xylene, and embedding in paraffin. Histological sections of 5 μm thickness were prepared using a microtome, stained with hematoxylin and eosin (H.E.), and mounted with coverslips. The prepared slides were examined under an Olympus light microscope equipped with a Moticam 1.3 MP digital camera (Motic Incorporation Ltd., Causeway Bay, Hong Kong).

### 2.8 Statistical Analysis

Statistical analyses were carried out using GraphPad Prism software, version 6.0. Data are expressed as mean ± standard deviation (SD) or standard error of the mean (SEM). Prior to analysis, the Shapiro–Wilk test was applied to verify data normality. For parameters measured only once per subject, one‐way ANOVA was used, with Bonferroni post hoc test for multiple comparisons. For parameters measured repeatedly in the same individuals over time (blood glucose level in OGTT), repeated‐measures ANOVA was performed, followed by Bonferroni post hoc test when appropriate. Differences were considered statistically significant at *p* < 0.05. For OGTT data, AUC (area under the curve) was calculated using GraphPad Prism for the raw response curves, with units corresponding to the Y‐axis values.

## 3 Results

### 3.1 Acute Toxicity Assessment

The acute toxicity test revealed that administration of WSMoL at 10, 25, and 50 mg/kg (i.p.) did not cause mortality or observable changes in behavior. No alterations in food intake, water consumption, or weight gain were observed in comparison with control, which received 0.15 M NaCl instead of lectin (Table 1). WSMoL administration at all doses led to monocytopenia (Table 2), with no other significant alterations in hematological parameters observed in comparison to the control group. As shown in Table 3, treatment with WSMoL at 10 and 25 mg/kg did not induce significant biochemical changes relative to the control group; however, a decrease in serum total protein levels was observed at the 50 mg/kg dose.

**TABLE 1:** Food intake, water consumption and weight gain of female Swiss albino mice treated with a single dose (10, 25, or 50 mg/kg; i.p.) of the water‐soluble lectin from *M. oleifera* seeds (WSMoL).

Parameters	Control	WSMoL (mg/kg)		
		10	25	50
Food intake (g/animal/day)	5.26 ± 0.12	6.60 ± 0.27	6.44 ± 0.28	6.44 ± 0.27
Water consumption (mL/animal/day)	6.80 ± 0.35	4.75 ± 0.07	4.98 ± 0.06	4.80 ± 0.11
Weight gain (g)	0.60 ± 0.74	0.80 ± 0.49	0.40 ± 0.42	0.60 ± 0.81

*Note:* Values represent mean ± standard deviation (*n* = 5/group). No significant differences (*p* > 0.05) were observed compared to the control.

**TABLE 2:** Hematological parameters of female Swiss albino mice treated with a single dose (10, 25 or 50 mg/kg; i.p.) of the water‐soluble lectin from *M. oleifera* seeds (WSMoL).

Parameters	Control	WSMoL (mg/kg)		
		10	25	50
Erythrocytes (10^6^/mm^3^)	11.9 ± 0.83	9.60 ± 0.30	12.1 ± 2.1	13.5 ± 0.39
Hematocrit (%)	49.6 ± 2.63	51.0 ± 1.7	50.1 ± 2.7	49.9 ± 2.63
Hemoglobin (g/dL)	17.2 ± 1.37	17.5 ± 1.33	17.7 ± 1.41	16.8 ± 2.51
MCV (fL)	53.8 ± 0.92	54.1 ± 0.87	53.6 ± 0.64	51.6 ± 0.62
MCH (pg)	16.3 ± 0.31	16.3 ± 0.18	16.2 ± 0.20	16.0 ± 0.62
MCHC (%)	30.3 ± 0.45	30.1 ± 0.52	30.1 ± 0.29	31.1 ± 1.37
Leukocytes (10^3^/mm^3^)	7.24 ± 0.95	5.28 ± 0.50	4.90 ± 0.51	6.12 ± 1.84
Segmented (%)	23.7 ± 2.19	31.8 ± 1.18	32.0 ± 1.53	21.0 ± 3.60
Lymphocytes (%)	67.7 ± 2.7	58.0 ± 0.0	59.7 ± 0.66	65.0 ± 1.73
Monocytes (%)	13.3 ± 0.66	6.0 ± 1.20^a^	6.75 ± 0.70^a^	5.0 ± 1.0^a^
Basophils (%)	0.20 ± 0.2	0.2 ± 0.2	0.0 ± 0.0	0.0 ± 0.0
Eosinophils (%)	2.20 ± 0.73	3.0 ± 0.94	2.0 ± 0.83	5.4 ± 2.23

*Note:* Values represent the mean ± standard deviation (*n* = 5/group).

Abbreviations: MCV: mean corpuscular volume; MCH: mean corpuscular hemoglobin; MCHC: mean corpuscular hemoglobin concentration.

^a^ Significant difference (*p* < 0.05) was observed in comparison with the control group.

**TABLE 3:** Blood biochemical parameters of female Swiss albino mice treated with a single dose (10, 25, or 50 mg/kg; i.p.) of the water‐soluble lectin from *M. oleifera* seeds (WSMoL).

Parameters	Control	WSMoL (mg/kg)		
		10	25	50
Total protein (g/dL)	6.57 ± 0.31	5.68 ± 0.12	6.67 ± 0.17	5.53 ± 0.12^a^
Cholesterol (mg/dL)	26.5 ± 1.98	24.2 ± 1.68	25.1 ± 0.84	23.5 ± 0.18
Triglycerides (mg/dL)	165.0 ± 15.2	164.0 ± 10.7	174.0 ± 7.03	178.0 ± 6.70
Glucose (mg/dL)	107.0 ± 1.33	131.0 ± 7.60	123.0 ± 4.33	126.0 ± 6.66
ALT (U/L)	13.4 ± 3.37	10.1 ± 0.76	7.04 ± 2.41	13.20 ± 7.24
AST (U/L)	86.5 ± 15.5	160.0 ± 20.9	101.0 ± 5.20	134.0 ± 11.0
GGT (U/dL)	20.9 ± 6.56	12.9 ± 3.72	16.3 ± 6.75	27.5 ± 7.45
Creatinine (mg/dL)	0.39 ± 0.16	0.47 ± 0.04	0.69 ± 0.55	0.61 ± 0.0
Urea (mg/dL)	6.10 ± 0.23	4.13 ± 1.0	3.85 ± 0.56	4.43 ± 0.79

*Note:* Values represent the mean ± standard deviation (*n* = 5/group).

Abbreviations: ALT: alanine aminotransferase; AST: aspartate aminotransferase; GGT: gamma‐glutamyl transferase.

^a^ Significant difference (*p* < 0.05) observed in comparison to the control group.

Single‐dose WSMoL treatments did not significantly alter the relative organ weights of the animals compared to the control group (Table 4). Histological analysis of liver tissues from all experimental groups (Figure 1) revealed a thin capsule composed of mesothelium and lobules containing veins of varying calibers, with parenchyma consisting of hepatocytes arranged irregularly around the central lobular vein. However, the groups treated with WSMoL at all doses exhibited a greater accumulation of mononuclear inflammatory infiltrates and hepatocytes with pyknotic nuclei compared to the control group.

**TABLE 4:** Relative organ weight (mg/g) of female Swiss albino mice treated with a single dose (10, 25 or 50 mg/kg; i.p.) of the water‐soluble lectin from *M. oleifera* seeds (WSMoL).

Organs	Control	WSMoL (mg/kg)		
		10	25	50
Liver	51.94 ± 1.34	50.22 ± 1.0	50.89 ± 0.93	54.59 ± 1.06
Spleen	6.15 ± 0.39	6.35 ± 0.54	5.52 ± 0.75	6.02 ± 0.23
Kidney	14.53 ± 1.19	13.22 ± 0.18	12.99 ± 0.61	13.0 ± 0.45

*Note:* Values represent mean ± standard deviation (*n* = 5/group). No significant differences (*p* > 0.05) were observed compared to the control.

**FIGURE 1:**
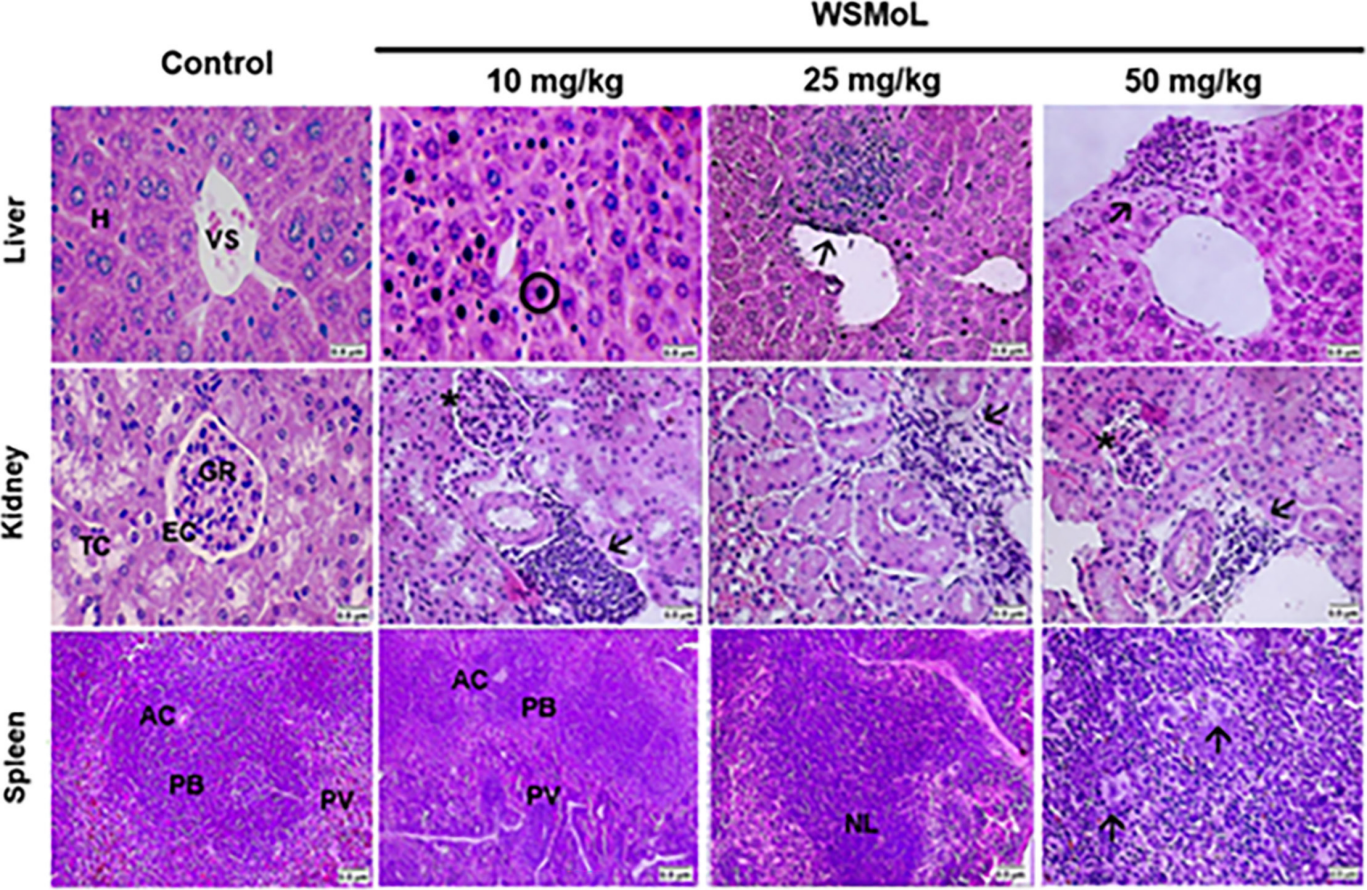
Representative photomicrograph of the liver, kidney and spleen of female Swiss albino mice treated with a single dose (10, 25, or 50 mg/kg; ip.) of the water‐soluble lectin from *M. oleifera* (WSMoL). Liver: Control group: hepatocytes (H) and blood vessels (VS) within normal limits; 10 mg/kg: Hepatocytes with pyknotic nuclei (circle); 25 and 50 mg/kg: perivascular inflammatory infiltrate (arrow). Kidneys: Control group: presence of renal glomeruli (GR), capsular space (EC) and convoluted tubules (TC); 10 mg/kg: mononuclear inflammatory infiltrate (arrow) and narrowing of the capsular space (asterisk); 25 mg/kg: mononuclear inflammatory infiltrate (arrow); 50 mg/kg: mononuclear inflammatory infiltrate (arrow) and increase of the capsular space (asterisk); Spleen: Control group: well‐defined red pulp (RP) and white pulp (BP). The white pulp is defined by the lymphoid nodule (NL) consisting of the germinal center and central arteriole (CA). 10 mg/kg: similar to the control; 25 mg/kg: disorganization of the lymphoid nodule with reduction of the central arteriole; 50 mg/kg: greater quantity of megakaryocytes. Approximation: 100× or 400×. Approximation 100×. H.E. staining. Scale bar: 0.5 μm.

The kidneys of animals in all experimental groups (Figure 1) exhibited a capsule covering the organ, along with well‐defined cortical and medullary regions. In the cortical region, renal glomeruli and proximal and distal convoluted tubules were observed. However, kidneys from animals treated with WSMoL at all doses showed the presence of mononuclear inflammatory infiltrates. Additionally, treatment with the 10 mg/kg dose resulted in a reduction of the subcapsular space, while administration of 50 mg/kg doses led to an increase in this space.

In all experimental groups, the spleen exhibited an external capsule covering the organ, with well‐defined red and white pulps (Figure 1). The white pulp contained lymphoid nodules consisting of a germinal center and a central arteriole. Animals treated with WSMoL at 10 mg/kg showed spleen morphology similar to that of the control group. However, treatment with WSMoL at doses of 25 and 50 mg/kg resulted in disorganization of the lymphoid nodules and a reduction in the central arteriole.

### 3.2 Evaluation of Repeated Dose Toxicity

Daily intraperitoneal administration of WSMoL at doses of 10, 25, or 50 mg/kg for 14 consecutive days in male and female mice did not cause mortality or alterations in behavior, body weight, food and water consumption (Table 5), or biochemical parameters (Table 6). Analysis of hematological data (Table 7) revealed a slight decrease in lymphocyte and monocyte counts in female mice treated with WSMoL at 50 mg/kg.

**TABLE 5:** Food intake, water consumption and weight gain of female and male Swiss albino mice treated for 14 days with repeated doses (10, 25, or 50 mg/kg i.p.) of the water‐soluble lectin from *M. oleifera* seeds (WSMoL).

Parameters	Female				Male			
	Control	WSMoL (mg/kg)			Control	WSMoL (mg/kg)		
		10	25	50		10	25	50
Food intake (g/animal/day)	4.18 ± 0.37	3.64 ± 0.36	3.95 ± 1.04	4.06 ± 0.40	6.83 ± 0.55	6.61 ± 1.00	6.10 ± 1.20	6.30 ± 0.91
Water consumption (mL/animal/day)	6.09 ± 0.33	6.27 ± 0.41	5.75 ± 0.80	6.43 ± 0.52	8.10 ± 0.15	7.90 ± 0.83	7.23 ± 1.33	7.49 ± 1.00
Weight gain (g)	0.60 ± 0.54	−0.20 ± 0.83	−0.22 ± 0.44	−0.60 ± 0.89	0.40 ± 0.89	0.60 ± 0.72	1.40 ± 1.51	0.80 ± 0.44

*Note:* Values represent mean ± standard deviation (*n* = 5/group). No significant differences (*p* > 0.05) were observed compared to the control.

**TABLE 6:** Blood biochemical parameters of female and male Swiss albino mice treated for 14 days with repeated doses (10, 25, or 50 mg/kg i.p.) of the water‐soluble lectin from *M. oleifera* seeds (WSMoL).

Parameters	Female				Male			
	Control	WSMoL (mg/kg)			Control	WSMoL (mg/kg)		
		10	25	50		10	25	50
Total Protein (g/dL)	3.81 ± 1.07	4.72 ± 0.43	5.95 ± 0.65	5.62 ± 0.29	4.76 ± 0.10	5.06 ± 0.23	6.04 ± 0.23	5.28 ± 0.30
Cholesterol (mg/dL)	22.2 ± 1.43	24.3 ± 1.07	20.4 ± 1.26	22.9 ± 1.46	24.0 ± 1.62	26.6 ± 1.65	26.6 ± 0.69	25.7 ± 0.62
Triglycerides (mg/dL)	179.0 ± 18.5	167.0 ± 5.57	162.0 ± 12.7	211.0 ± 12.8	152.0 ± 7.96	168.0 ± 13.6	166.0 ± 3.87	172.0 ± 6.0
Glucose (mg/dL)	123.0 ± 5.04	128.0 ± 2.14	128.0 ± 5.81	121.0 ± 1.55	126.0 ± 2.44	139.0 ± 6.67	125.0 ± 5.79	136.0 ± 5.48
ALT (U/L)	16.1 ± 2.05	14.1 ± 3.31	8.36 ± 1.27	7.34 ± 1.55	11.9 ± 3.30	5.50 ± 0.91	8.81 ± 0.51	14.3 ± 5.25
AST (U/L)	137.0 ± 27.4	99.1 ± 41.1	116.0 ± 54.4	103.0 ± 19.6	94.5 ± 11.90	141.0 ± 30.3	103.0 ± 18.2	95.20 ± 26.5
GGT (mg/dL)	5.80 ± 5.60	6.44 ± 0.74	7.22 ± 2.63	10.3 ± 2.58	11.2 ± 0.43	7.09 ± 1.86	8.59 ± 2.15	8.12 ± 0.63
Creatinine (mg/dL)	0.54 ± 0.27	0.15 ± 0.04	0.27 ± 0.07	0.48 ± 0.05	0.53 ± 0.19	0.11 ± 0.01	0.58 ± 0.05	0.36 ± 0.12
Urea (mg/dL)	5.39 ± 1.34	5.50 ± 0.67	4.17 ± 0.79	6.30 ± 0.52	5.79 ± 0.66	5.02 ± 0.83	5.69 ± 1.92	4.71 ± 0.96

*Note:* Values represent mean ± standard deviation (*n* = 5/group). No significant differences (*p* > 0.05) were observed compared to the control.

Abbreviations: ALT, alanine aminotransferase; AST, aspartate aminotransferase; GGT, gamma‐glutamyl transferase.

**TABLE 7:** Hematological parameters of female and male Swiss albino mice treated with repeated doses (10, 25, and 50 mg/kg i.p.) of the water‐soluble lectin from *M. oleifera* seeds (WSMoL).

Parameters	Female				Male			
	Control	WSMoL (mg/kg)			Control	WSMoL (mg/kg)		
		10	25	50		10	25	50
Erythrocytes (10^6^/mm^3^)	5.45 ± 0.75	5.88 ± 0.27	5.11 ± 0.67	5.23 ± 0.34	5.48 ± 0.96	5.34 ± 0.53	6.46 ± 0.01	6.94 ± 0.09
Hematocrit (%)	33.2 ± 3.19	27.3 ± 0.87	22.0 ± 0.36	26.4 ± 2.21	47.7 ± 3.1	45.9 ± 1.23	43.6 ± 0.51	42.2 ± 0.88
Hemoglobin (g/dL)	11.7 ± 1.01	9.74 ± 0.54	9.78 ± 1.18	9.68 ± 0.91	15.1 ± 1.09	14.3 ± 0.29	13.7 ± 0.23	13.0 ± 0.22
MCV (fL)	49.5 ± 0.75	47.42 ± 0.55	47.05 ± 0.66	50.38 ± 0.92	52.3 ± 0.61	52.5 ± 0.64	52.1 ± 0.15	52.1 ± 0.62
MCH (pg)	15.8 ± 0.43	16.5 ± 0.16	19.1 ± 0.21	17.68 ± 0.39	16.8 ± 0.14	16.3 ± 0.07	16.1 ± 0.31	17.0 ± 0.23
MCHC (%)	35.2 ± 0.16	35.6 ± 0.10	37.4 ± 0.53	36.6 ± 0.38	31.6 ± 0.35	31.2 ± 0.47	31.0 ± 0.59	32.1 ± 0.81
Leukocytes (10^3^/mm^3^)	3.1 ± 0.52	3.79 ± 0.83	3.6 ± 0.66	4.8 ± 1.26	1.63 ± 0.16	1.45 ± 0.16	1.47 ± 0.22	1.58 ± 0.11
Segmented (%)	34.3 ± 2.19	35.0 ± 2.0	37.3 ± 1.86	34.3 ± 1.76	33.3 ± 3.25	31.3 ± 0.33	33.7 ± 2.33	33.3 ± 3.53
Lymphocytes (%)	51.8 ± 2.56	44.3 ± 2.60	56.2 ± 1.53	45.0 ± 2.0^a^	58.7 ± 0.33	57 ± 2.08	58.0 ± 2.0	59.0 ± 2.08
Monocytes (%)	13.8 ± 3.06	10.2 ± 3.68	6.6 ± 2.25	7.6 ± 0.93^a^	5.61 ± 1.67	3.0 ± 0.58	2.57 ± 0.33	5.33 ± 2.03
Basophils (%)	0.22 ± 1.59	0.4 ± 0.40	0.6 ± 0.40	0.62 ± 0.51	0.33 ± 0.33	0.0 ± 0.0	0.0 ± 0.0	0.0 ± 0.0
Eosinophils (%)	4.40 ± 1.69	1.6 ± 0.40	2.0 ± 0.71	5.6 ± 1.96	1.67 ± 0.33	2.67 ± 0.33	4.0 ± 0.58	5.67 ± 1.76

*Note:* Values represent mean ± standard deviation (*n* = 5/group).

Abbreviations: MCV: mean corpuscular volume; MCH: mean corpuscular hemoglobin; MCHC: mean corpuscular hemoglobin concentration.

^a^ Significant difference (*p* < 0.05) observed in comparison to the control group.

Histological analysis (Figure 2) revealed that the livers of all animals exhibited a thin external capsule composed of mesothelium and parenchyma, with hepatocytes arranged irregularly around the central lobular vein. Animals treated with WSMoL at all doses showed mononuclear inflammatory infiltrates. In the kidneys of all groups, a capsule covering the organ and well‐defined cortical and medullary regions were observed, including renal glomeruli and proximal and distal convoluted tubules. However, animals treated with WSMoL showed mononuclear inflammatory infiltrates and renal cells with pyknotic nuclei. Additionally, the 50 mg/kg dose caused a reduction in the subcapsular space compared to other groups, particularly in females. The spleen exhibited a capsule covering the organ and well‐defined red and white pulps, but disorganization of the lymphoid nodules with a reduction in the central arteriole was observed in WSMoL‐treated animals. Table 8 presents the relative organ weights of mice treated daily with WSMoL for 14 days. A significant reduction in kidney weight was observed only in female mice treated with doses of 50 and 25 mg/kg.

**FIGURE 2:**
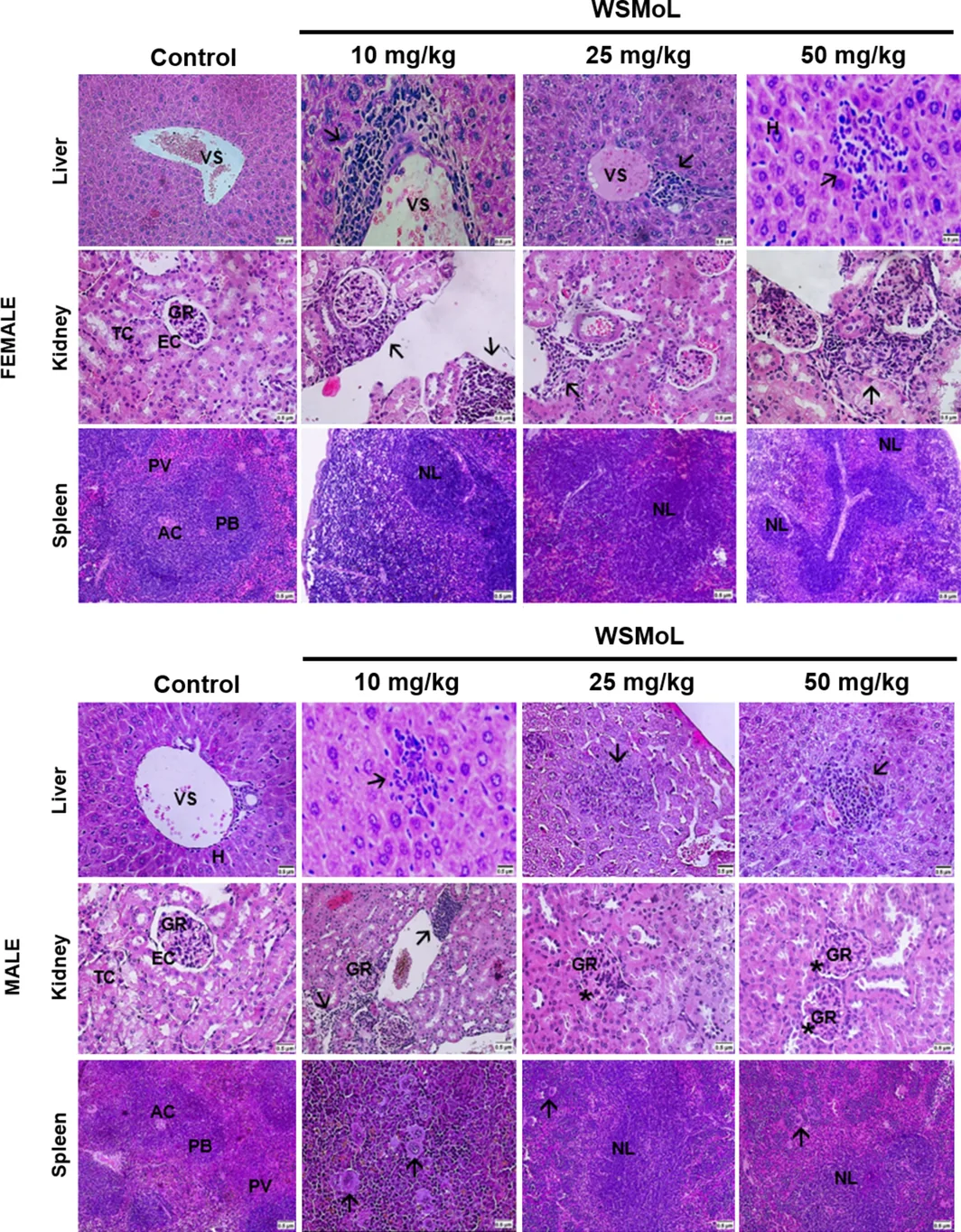
Representative photomicrographies of liver, kidney, and spleen from female and male Swiss albino mice treated with a 14‐day repeated doses (10, 25, or 50 mg/kg) of water‐soluble lectin from *M. oleifera* (WSMoL). FEMALES—Liver: Control group: blood vessels (BV) within normal limits; 10 and 25 mg/kg: perivascular infiltrate (arrow); 50 mg/kg: Hepatocytes (H) and inflammatory infiltrate located in the liver parenchyma (arrow); Kidney: Control group: kidney from the control group observing the presence of renal glomeruli (RG), capsular space (CS), and convoluted 5tubules (Ct); 10, 25, and 10 mg/kg: mononuclear infiltrate (arrow); Spleen: Control group: red pulp (RP) and well‐defined white pulp (WP). The white pulp is defined by the lymphoid nodule (LN) consisting of the germinal center and central arteriole (CA); 10, 25, and 50 mg/kg: disorganization of the lymphoid nodule with reduction of the central arteriole. MALES—Liver: Control group: hepatocytes (H) and blood vessels (BV) within normal limits; 10 and 25 mg/kg: inflammatory infiltrate located in the liver parenchyma (arrow); 50 mg/kg: perivascular infiltrate (arrow). Kidney: Control group: kidney from the control group showing the presence of renal glomeruli (RG), capsular space (CS), and convoluted tubules (Ct); 10 mg/kg: mononuclear inflammatory infiltrate (arrow); 25 and 50 mg/kg: reduced capsular space (asterisk). Spleen: Control group: red pulp (RP) and well‐defined white pulp (WP). The white pulp is defined by the lymphoid nodule (LN) consisting of the germinal center and central arteriole (CA). 10 mg/kg: a greater presence of megakaryocytes (arrows). 25 and 50 mg/kg: disorganization of the lymphoid nodule with reduction of the central arteriole and presence of megakaryocytes (arrows). Approximation: 100× or 400×; H.E. staining; Scale bar: 0.5 μm.

**TABLE 8:** Relative organ weights of female and male Swiss mice treated with repeated doses (10, 25, or 50 mg/kg i.p.) of the water‐soluble lectin from *M. oleifera* seeds (WSMoL).

Organs	Female				Male			
	Control	WSMoL (mg/kg)			Control	WSMoL (mg/kg)		
		10	25	50		10	25	50
Liver	42.7 ± 2.12	51.65 ± 2.44	45.21 ± 3.13	50.27 ± 2.49	62.47 ± 1.37	64.75 ± 2.13	63.82 ± 0.56	59.5 ± 3.30
Spleen	8.35 ± 0.46	7.76 ± 0.80	8.40 ± 0.22	9.19 ± 0.74	8.35 ± 0.70	7.77 ± 0.82	9.48 ± 0.33	9.87 ± 0.78
Kidney	16.10 ± 0.95	13.58 ± 0.98	12.67 ± 0.39^a^	12.74 ± 0.34^a^	16.21 ± 0.48	14.24 ± 0.72	15.67 ± 0.39	15.81 ± 0.94

*Note:* Values represent mean ± standard deviation (*n* = 5/group).

^a^ Significant difference (*p* < 0.05) observed in comparison to the control group.

### 3.3 Genotoxicity

The data from the micronucleus assay showed no significant difference in the number of micronucleated polychromatic erythrocytes (MnPCE) in animals treated with WSMoL at any dose and those in the negative control group (Figure 3). As expected, the positive control group (treated with methotrexate) showed a significantly (*p* < 0.0001) higher number of MnPCE.

**FIGURE 3:**
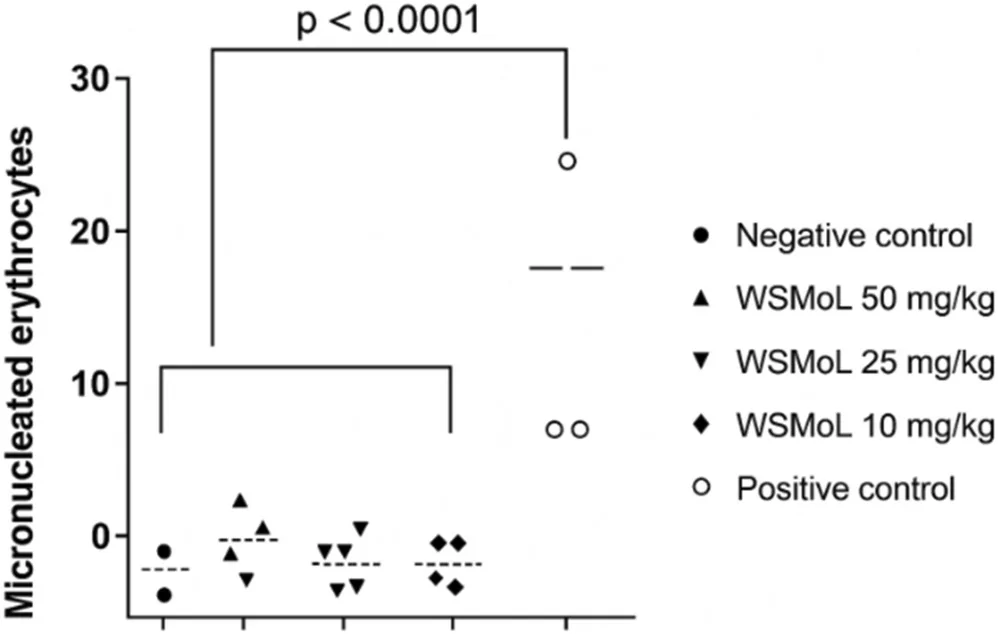
Number of micronucleated polychromatic erythrocytes in bone marrow cells of female Swiss albino mice treated with different doses (10, 25, or 50 mg/kg i.p.) of water‐soluble lectin from *M. oleifera* seeds (WSMoL), methotrexate (positive control) or 0.15 M NaCl (negative control).

### 3.4 Hypoglycemic Activity of WSMoL in Alloxan‐Treated Mice

The effect of intraperitoneal administration of WSMoL at 25 and 50 mg/kg was evaluated in male and female mice with sustained hyperglycemia pharmacologically induced by alloxan monohydrate (fasting glucose > 400 mg/dL). Table 9 presents data on body weight gain, water consumption, and food intake. A significant reduction in weight gain was observed in both male and female diabetic mice treated with 0.15 M NaCl (negative control) compared to the nondiabetic (Sham) group. The animals treated with insulin (3 units/day) or WSMoL (25 and 50 mg/kg) still presented a weight loss, but to a significantly (*p* < 0.05) lower extent than the negative control group.

**TABLE 9:** Weight gain, food intake and water consumption of normoglycemic (Sham) or hyperglycemic mice subjected to intraperitoneal treatment for 10 days with water‐soluble lectin from *M. oleifera* seeds (WSMoL, 25, or 50 mg/kg), saline solution (Sham and negative control) or insulin (positive control, 3 units/day).

Groups	Females			Males		
	Weight gain (g)	Food intake (g/animal/day)	Water consumption (mL/animal/day)	Weight gain (g)	Food intake (g/animal/day)	Water consumption (mL/animal/day)
Sham	0.0 ± 1.0	5.3 ± 0.31	6.18 ± 0.15	2.0 ± 1.00	4.32 ± 0.23	6.22 ± 0.15
Negative control	−11.75 ± 1.4^a^	10.5 ± 0.29^a^	12.6 ± 0.22^a^	−3.75 ± 0.55^a^	10.7 ± 0.24^a^	12.5 ± 0.33^a^
Positive control	−2.4 ± 0.89^a^, ^b^	6.2 ± 0.32^b^	7.18 ± 0.85^b^	−0.1 ± 0.71^a^, ^b^	5.2 ± 0.33^b^	6.4 ± 0.29^b^
WSMoL 25 mg/kg	−2.6 ± 0.55^a^, ^b^	7.5 ± 0.63^a^, ^b^	9.36 ± 1.87^a^, ^b^	−0.8 ± 0.44^a^ ^,b^	7.58 ± 0.82^a^, ^b^	7.98 ± 1.05^a^ ^,b^
WSMoL 50 mg/kg	−3.4 ± 0.9^a^, ^b^	7.94 ± 0.71^a^, ^b^	9.14 ± 1.69^a^, ^b^	−2.0 ± 0.71^a^, ^b^	8.34 ± 0.85^a^, ^b^	8.62 ± 0.87^a^, ^b^

*Note:* Values are represented by mean ± standard deviation (*n* = 5/group).

^a^ Significant difference (*p* < 0.05) compared to the Sham group.

^b^ Significant difference (*p* < 0.05) compared to the negative control group.

Water consumption in the negative control group was 12.6 ± 0.22 (females) and 12.5 ± 0.33 (males) mL/animal/day, compared to 6.18 ± 0.15 (females) and 6.22 ± 0.15 (males) mL/animal/day in the Sham group, indicating that hyperglycemia resulted in polydipsia. Polyphagia was also observed since food intake in the negative control group was 10.5 ± 0.29 (females) and 10.7 ± 0.24 (males) g/animal/day, compared to 5.3 ± 0.31 (females) and 4.32 ± 0.23 (males) g/mL/animal/day in the Sham group. Table 9 also shows that all hyperglycemic animals treated with WSMoL consumed significantly (p < 0.05) more food and water compared to the normoglycemic group (Sham). On the other hand, the insulin‐treated group showed no significant differences from the Sham group. The animals treated with WSMoL exhibited lower (*p* < 0.05) food and water intake than the negative control group. These effects on food and water consumption, as well as weight gain, were consistent for both male and female animals.

Treatment with WSMoL (25 and 50 mg/kg) resulted in a significant reduction in blood glucose levels in both hyperglycemic male and female mice (Figure 4). In male mice, blood glucose levels decreased significantly to approximately 105 mg/dL following administration of either WSMoL dose, reaching values comparable to those observed in the insulin‐treated group. In contrast, animals in the negative control maintained extremely high glucose levels (~450 mg/dL). In female mice, the 25 mg/kg dose of WSMoL was less effective (120 mg/dL) than the 50 mg/kg dose (105 mg/dL). The higher dose produced a hypoglycemic effect statistically comparable to insulin.

**FIGURE 4:**
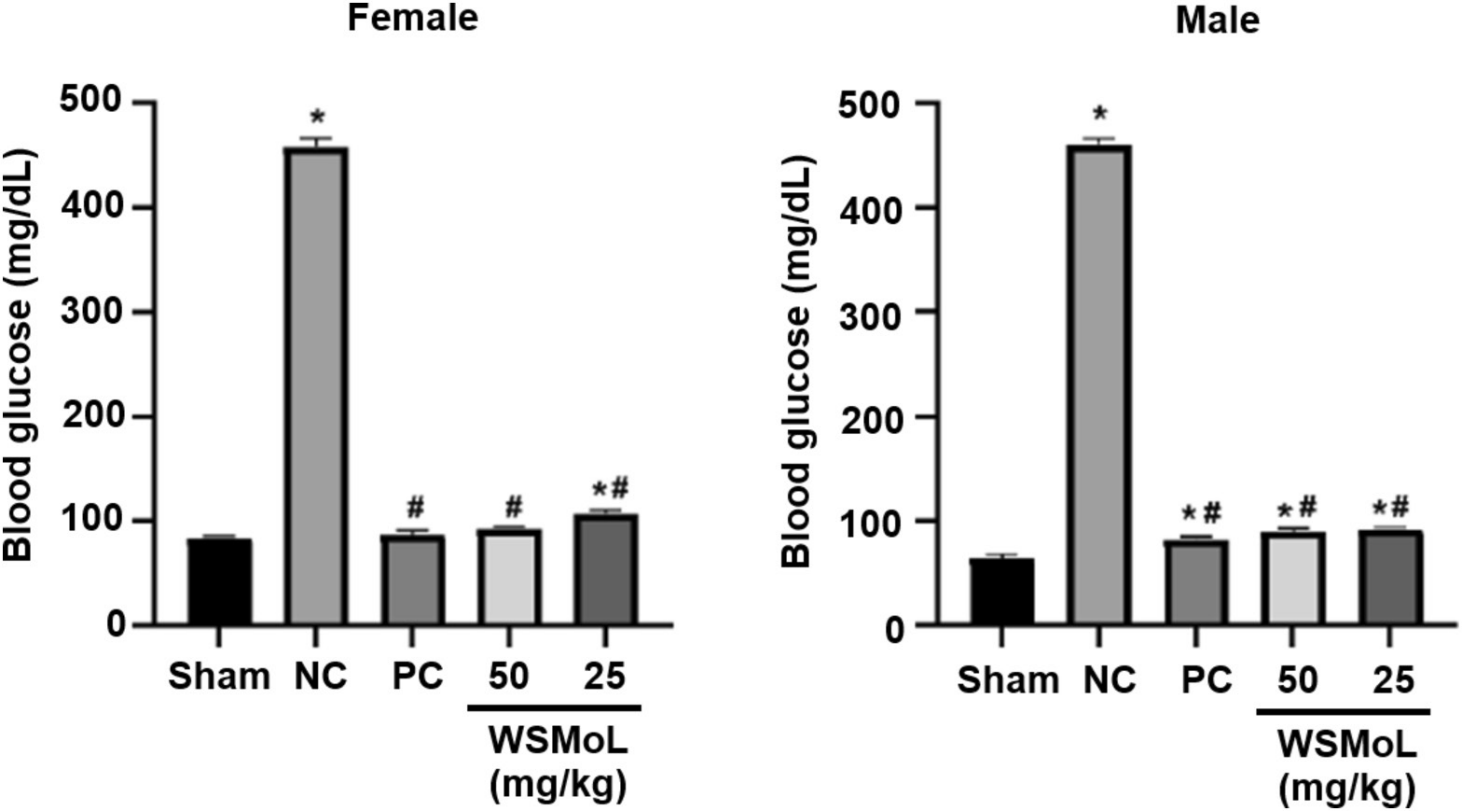
Blood glucose ofnormoglycemic (Sham) or hyperglycemic mice subjected to intraperitoneal treatment for 10 days with water‐soluble lectin from *M. oleifera* seeds (WSMoL, 25, or 50 mg/kg), saline solution (Sham and negative control, NC) or insulin (positive control, PC, 3 units/day). The bars represent mean ± standard error of the mean (SEM) (*n* = 5/group). (*) Significant difference (*p* < 0.05) compared to the Sham group. (#) Significant difference (*p* < 0.05) compared to the negative control.

OGTT data are presented in Table 10. In animals treated with WSMoL (25 and 50 mg/kg), insulin, and the Sham group, blood glucose levels returned to baseline (time 0) after 120 min. In contrast, animals in the negative control group maintained elevated and stable blood glucose levels (~600 mg/dL) throughout the test, confirming both the impairment of glycemic response and the effectiveness of the hyperglycemic model. The 50 mg/kg WSMoL dose was more effective overall, reducing blood glucose by 57.7% (from 221.4 to 93.6 mg/dL at 120 min) in males and by 62.8% (from 248.6 to 92.4 mg/dL) in females. The 25 mg/kg dose was also effective in males and females, achieving 61.0% and 58.8% reduction over the same period, respectively.

**TABLE 10:** Oral glucose tolerance test (OGTT) in normoglycemic (Sham) or hyperglycemic mice subjected to intraperitoneal treatment for 10 days with water‐soluble lectin from *M. oleifera* seeds (WSMoL, 25, or 50 mg/kg), saline solution (Sham and negative control) or insulin (positive control, 3 units/day).

Groups	Blood glucose (mg/dL)				AUC
	0 min	30 min	60 min	120 min	
	Females				
Sham	83.8 ± 5.45	199.6 ± 7.82	93.2 ± 4.02	82.0 ± 6.08	13,899 ± 293.1
Negative control	458.8 ± 18.8^a^	600.0 ± 0.0	600.0 ± 0.0	600.0 ± 0.0	69,882 ± 282.0^a^
Positive control	86.8 ± 9.61^b^	200.0 ± 8.81^b^	93.6 ± 4.16^b^	86.6 ± 5.18^b^	14,112 ± 315.2^b^
WSMoL 25 mg/kg	92.6 ± 4.51^b^	248.6 ± 21.48^a^, ^b^	183.2 ± 7.85^a^, ^b^	92.4 ± 7.46^b^	19,863 ± 575.9^b^
WSMoL 50 mg/kg	107.2 ± 7.66^a^, ^b^	279.6 ± 15.76^a^, ^b^	183.2 ± 15.4^a^, ^b^	115.0 ± 6.88^a^, ^b^	21,690 ± 659.1^b^
	Males				
Sham	64.6 ± 7.64	184.4 ± 7.22	76.2 ± 10.15	66.4 ± 5.03	11,922 ± 418.6
Negative control	461.0 ± 12.90^a^	600.0 ± 0.0	600.0 ± 0.0	600.0 ± 0.0	69,915 ± 193.5^a^
Positive control	82.8 ± 6.75^b^	201.4 ± 10.4^b^	92.6 ± 4.49^b^	84.0 ± 5.34^a,^ ^b^	13,971 ± 327.5^b^
WSMoL 25 mg/kg	90.0 ± 7.37^a^, ^b^	221.4 ± 12.4^a^, ^b^	142.0 ± 27.0^a^, ^b^	93.6 ± 5.68^a^, ^b^	17,190 ± 964.7^b^
WSMoL 50 mg/kg	92.2 ± 4.65^b^	278.4 ± 10.4^a,^ ^b^	171.2 ± 17.2^a^, ^b^	108.4 ± 7.13^a^, ^b^	20,691 ± 657.4^b^

*Note:* Values are represented by the mean ± standard deviation (*n* = 5/group). AUC: area under curve calculated for the raw response curves.

^a^ Significant difference (*p* < 0.05) compared to the Sham group.

^b^ Significant difference (*p* < 0.05) compared to the negative control group.

Additional biochemical parameters for hyperglycemic and normoglycemic animals are presented in Table 11. In both sexes, hepatic and renal markers were significantly elevated in hyperglycemic animals compared to the normoglycemic (Sham) group. The treatment with WSMoL at 25 and 50 mg/kg significantly improved these markers in both male and female mice, with values differing markedly (*p* < 0.05) from the negative control.

**TABLE 11:** Blood biochemical parameters of normoglycemic (Sham) or hyperglycemic mice subjected to intraperitoneal treatment for 10 days with water‐soluble lectin from *M. oleifera* seeds (WSMoL, 25, or 50 mg/kg), saline solution (Sham and negative control) or insulin (positive control, 3 units/day).

Parameters	Females				
	Sham	Negative control	Positive control	WSMoL (mg/kg)	
				25	50
Total Protein (g/dL)	6.5 ± 0.22	5.1 ± 0.44^a^	6.2 ± 0.20^b^	5.9 ± 0.67	6.2 ± 0.22^b^
Albumin (g/dL)	3.7 ± 0.22	3.6 ± 0.17	3.6 ± 0.17	3.7 ± 0.08	3.3 ± 0.02
Cholesterol (mg/dL)	97.2 ± 2.45	95.4 ± 2.45	93.6 ± 2.46	85.6 ± 4.24	86.2 ± 3.8
ALT (U/L)	61.94 ± 1.34	100.3 ± 2.63^a^	82.93 ± 2.35^a^, ^b^	77.7 ± 6.01^a^ ^,b^	74.18 ± 7.04^a^ ^,b^
AST (U/L)	130 ± 2.23	227 ± 2.01^a^	172 ± 13.86^a^ ^,b^	125 ± 7.15^b^	115 ± 4.91^a^ ^,b^
GGT (mg/dL)	14.3 ± 0.20	20.5 ± 1.34^a^	15.6 ± 1.78^,b^	12.9 ± 0.44^b^	12.5 ± 0.89^b^
Urea (mg/dL)	6.37 ± 0.02	12.5 ± 0.44^a^	8.1 ± 0.13^a^ ^,b^	11.7 ± 1.56^a^	9.3 ± 0.22^a^ ^,b^
Creatine (mg/dL)	0.4 ± 0.13	0.9 ± 0.06^a^	0.6 ± 0.04^b^	0.5 ± 0.06^b^	0.5 ± 0.06^b^
Parameters	Males				
	Sham	Negative control	Positive control	WSMoL (mg/kg)	
				25	50
Total Protein (g/dL)	5.93 ± 0.44	6.4 ± 0.67	6.2 ± 0.22	6.6 ± 0.47	6.3 ± 0.89
Albumin (g/dL)	3.83 ± 0.44	3.7 ± 0.22	3.8 ± 0.20	3.73 ± 0.11	3.8 ± 0.17
Cholesterol (mg/dL)	95.3 ± 2.41	107 ± 10.06	101 ± 3.80	90.5 ± 4.47^b^	86.3 ± 5.14^b^
ALT (U/L)	65.0 ± 1.34	97.2 ± 6.39^a^	88.5 ± 4.38^a^	86.5 ± 6.48^a^	84.9 ± 8.72^a^, ^b^
AST (U/L)	96.5 ± 3.80	210 ± 7.82^a^	168 ± 11.62^a^, ^b^	144 ± 12.96^a^, ^b^	128 ± 4.47^a^, ^b^
GGT (mg/dL)	9.49 ± 0.22	15.3 ± 1.34^a^	13.8 ± 0.89^a^, ^b^	13.5 ± 0.44^a^, ^b^	12.9 ± 0.22^a^, ^b^
Urea (mg/dL)	6.1 ± 0.20	14.6 ± 0.89^a^	14.5 ± 2.01^a^	10.2 ± 0.48^a^, ^b^	10.5 ± 1.11^a^, ^b^
Creatine (mg/dL)	0.4 ± 0.06	1.04 ± 0.06^a^	0.8 ± 0.11^a^	0.5 ± 0.09^b^	0.5 ± 0.06^b^

*Note:* Values are represented by the mean ± standard deviation (*n* = 5/group).

Abbreviations: ALT: alanine aminotransferase; AST: aspartate aminotransferase; GGT: gamma‐glutamyl transferase.

^a^ Significant difference (*p* < 0.05) compared to the Sham group.

^b^ Significant difference (*p* < 0.05) compared to the negative control group.

As shown in Table 12, no significant differences were observed in the relative organ weights of male and female animals treated with insulin or WSMoL (25 and 50 mg/kg) compared to the Sham group. However, the liver weight of animals in the negative control group was significantly higher, differing from all other groups.

**TABLE 12:** Relative organ weights of normoglycemic (Sham) or hyperglycemic mice subjected to intraperitoneal treatment for 10 days with water‐soluble lectin from *M. oleifera* seeds (WSMoL, 25, or 50 mg/kg), saline solution (Sham and negative control) or insulin (positive control, 3 units/day).

Groups	Female		Male	
	Liver	Kidney	Liver	Kidney
Sham	54.32 ± 8.13	13.81 ± 2.75	54.55 ± 2.61	16.61 ± 0.80
Negative control	79.9 ± 2.95^a^	16.98 ± 4.20	85.89 ± 14.17^a^	21.46 ± 0.71
Positive control	58.02 ± 9.05^b^	14.96 ± 2.45	50.72 ± 9.50^b^	17.9 ± 2.25
WSMoL 25 mg/kg	52.56 ± 6.46^b^	15.41 ± 2.54	46.9 ± 14.4^b^	16.13 ± 3.80
WSMoL 50 mg/kg	61.8 ± 5.03^b^	16.52 ± 2.12	59.6 ± 3.39^b^	15.25 ± 4.06

*Note:* Values are represented by mean ± standard deviation (*n* = 5/group).

^a^ Significant difference (*p* < 0.05) compared to the Sham group.

^b^ Significant difference (*p* < 0.05) compared to the control group.

Histological analysis of male and female animals (Figure 5) revealed that the negative control group exhibited hepatic inflammatory infiltrates and cytoplasmic vacuolation, along with glomerular damage, reduced capsular space, and tubular obstruction in the kidneys. In contrast, animals treated with insulin or WSMoL (25 and 50 mg/kg) showed only mild hepatic cytoplasmic vacuolation and minimal inflammatory infiltrates. The kidneys of insulin‐treated animals appeared histologically normal. However, male and female mice treated with WSMoL at both doses displayed slight alterations in the capsular space and renal tubules, suggesting partial resolution of tissue damage.

**FIGURE 5:**
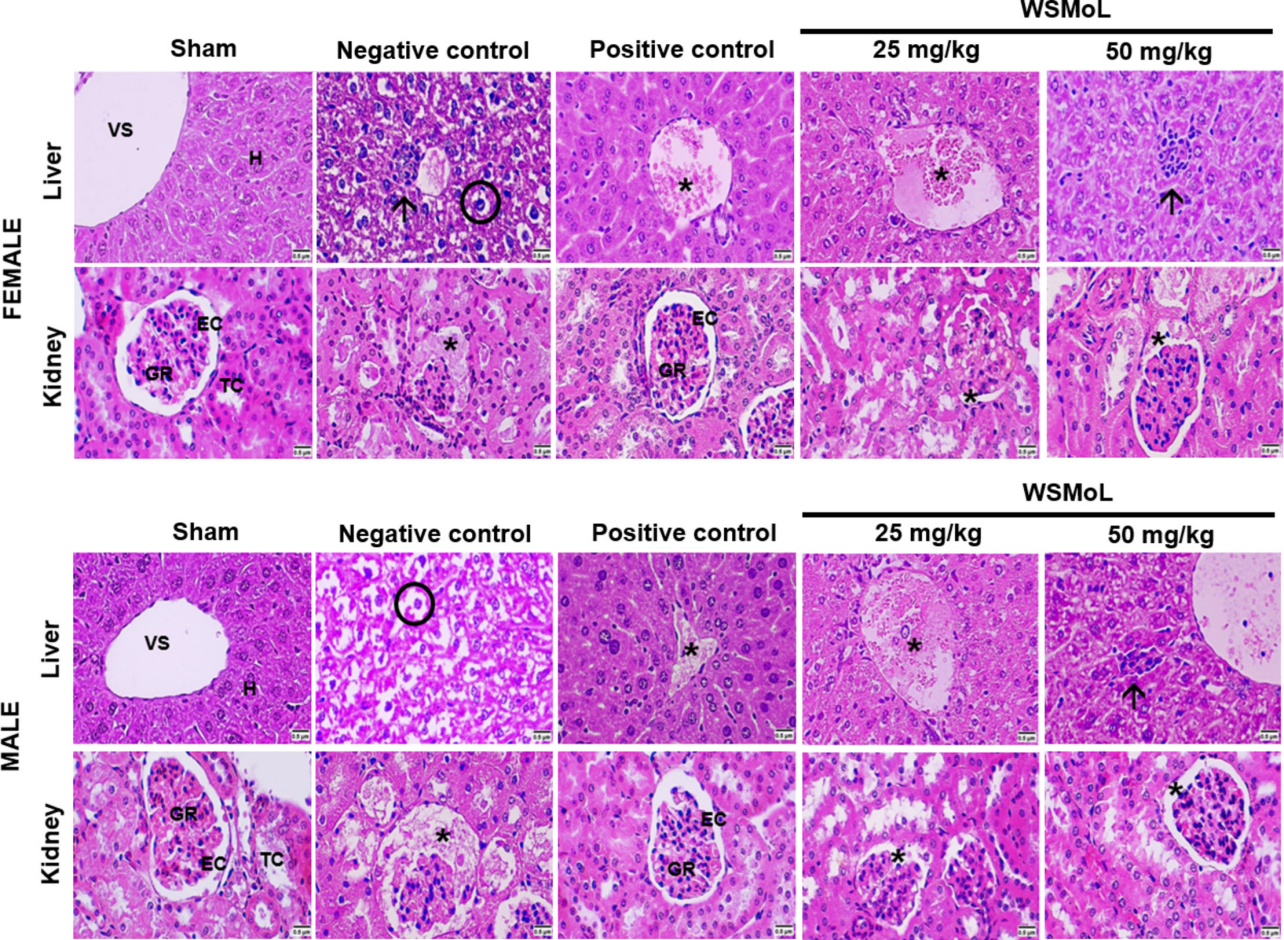
Representative photomicrographies of the liver and kidney of normoglycemic (Sham) or hyperglycemic mice subjected to intraperitoneal treatment for 10 days with water‐soluble lectin from *M. oleifera* seeds (WSMoL, 25, or 50 mg/kg), saline solution (Sham and negative control) or insulin (positive control, 3 units/day). Liver—Sham: hepatocytes (H) and blood vessels (VS) within normal limits; Negative control: inflammatory infiltrate (arrow) and cytoplasmic vacuolation (circle); Insulin: hepatocytes within normal limits and vessels with congestion (star); 25 mg/kg: mild cytoplasmic vacuolation and congestion (star); 50 mg/kg: mild cytoplasmic vacuolation, congestion, and presence of brief inflammatory infiltrates (arrow). Kidneys—Sham: Renal cortex showing glomerulus (GR), capsular space (EC), and convoluted tubules (TC) within normal limits; Negative control: renal cortex showing glomerulus with apparent glomerular damage, compromised capsular space (asterisk); Insulin: Structures within normal limits; 25 mg/kg: Glomerulus with apparent glomerular damage and compromised capsular space (star). 50 mg/kg: Glomerulus with glomerular damage and compromised capsular space (star); Approximation: 400×; H.E. staining; Scale bar: 0.5 μm.

## 4 Discussion

In this study, we evaluated the potential hypoglycemic activity of WSMoL using a T1DM‐like model induced by alloxan in mice, as well as its possible ameliorative effects on the function of key organs commonly affected by diabetic complications. However, Brito et al. (2023) reported that animals treated intraperitoneally with WSMoL at 100 mg/kg exhibited decreased food and water intake and reduced weight gain, whereas 60% of those receiving a 200 mg/kg dose did not survive. Therefore, we first conducted a toxicity assessment, including both acute and 14‐day repeated dose studies, prior to performing the pharmacological evaluation of its hypoglycemic effect.

Hippocratic screening is commonly employed in the preliminary evaluation of plant‐derived compounds to identify potential toxic effects (Jesus et al. 2012). At the doses investigated in the present study, animals treated with WSMoL did not exhibit any behavioral signs of intoxication, in contrast to the observations reported by Brito et al. (2023) for WSMoL administered at 100 or 200 mg/kg, which included decreased ambulation, reddish tail, drowsiness, constipation, and abdominal spasms.

The toxicity assessment proceeded with the evaluation of hematological and biochemical parameters. The monocytopenia observed in female mice in both the acute and repeated‐dose toxicity assays, as well as the lymphocytopenia detected in the repeated‐dose test, may be related to the immunomodulatory effects previously reported for WSMoL (Coriolano et al. 2018) and potentially to the ability of this lectin to interfere with the proliferation of monocytic and lymphocytic progenitors in the bone marrow. Notably, monocytopenia and leukocytopenia were not observed in animals treated with WSMoL at 100 mg/kg (Brito et al. 2023), suggesting that the immunomodulatory effects of the lectin are triggered by lower doses.

Assessment of biochemical parameters in mouse blood can reveal alterations across multiple physiological systems, providing insights into the function of various organs and tissues (Silva‐Santana et al. 2020). In the present study, the only notable change observed was a decrease in serum total protein levels in animals that received a 50 mg/kg dose of WSMoL in the acute toxicity assay. These results indicate that the mice tolerated the administered doses of WSMoL well, and the observed decrease in serum total protein appears to be a transient, dose‐specific effect without lasting impact on systemic function.

Histological analysis of WSMoL‐treated animals primarily revealed inflammatory infiltrates in the liver and kidneys, as well as a splenic response characterized by alterations in the organization of lymphoid nodules. These findings collectively suggest an inflammatory reaction to WSMoL, further supported by the presence of pyknotic nuclei, indicative of cellular stress and/or apoptosis (Hou et al. 2016; Lira et al. 2023). Also, the reduction in the kidney subcapsular space observed in the histological analysis is likely correlated with this inflammatory response, being a consequence of immune cell infiltration (Tanaka 2024).

As a plant‐derived lectin, WSMoL is a foreign protein that can be recognized by the immune system as a potential antigen, triggering innate immune responses, including the recruitment of inflammatory cells to target organs. Thus, the observed splenic alterations may reflect a compensatory response to systemic immune activation. The inflammatory response observed in WSMoL‐treated animals may be associated with the toxicity signs reported by Brito et al. (2023) at higher, and in some cases lethal, doses of this protein. These findings raise concerns about the potential for chronic inflammation associated with prolonged use, warranting further investigation in future studies.

Following the completion of the toxicity assessment, the hypoglycemic activity of WSMoL was evaluated using doses of 25 and 50 mg/kg because they were sub‐lethal and did not cause major hematological/biochemical alterations, despite inducing localized tissue inflammation. Given the histological alterations detected during the toxicity study, animals in the hypoglycemic assay were also subjected to histological analysis at the end of the experiment. This approach allowed not only the monitoring of potential tissue effects of WSMoL at pharmacologically relevant doses but also the evaluation of possible protective or ameliorative effects against organ damage induced by the T1DM‐like condition triggered by alloxan.

As shown in the results, administration of WSMoL reduced blood glucose levels, improved oral glucose tolerance, and alleviated polyphagia and polydipsia, which are hallmark clinical manifestations of diabetes mellitus (DeFronzo et al. 2015). The data further suggest that WSMoL may enhance peripheral glucose utilization in treated animals, likely through mechanisms related to modulation of glucose uptake or insulin sensitivity, as reported for other hypoglycemic plant‐derived proteins (Simha et al. 2024). Overall, the effects of WSMoL were comparable in male and female mice, with some variations likely reflecting intrinsic physiological and hormonal differences between sexes, such as disparities in insulin sensitivity, metabolic rates, or the expression of glucose receptors and transporters (Mauvais‐Jarvis 2018).

Elevated serum levels of AST and ALT in diabetic mice indicate that alloxan induced liver damage and compromised the functional integrity of hepatocyte membranes (Lucchesi et al. 2015). Diabetes is also commonly associated with renal damage, characterized by glomerular and tubular cell hypertrophy, matrix expansion, and increased renal blood flow, leading to functional impairment. Moreover, high urea levels and renal hypertrophy in diabetic animals are caused by increased formation of advanced glycation end products and accumulation of glycogen granules in the distal tubules (Yankuzo et al. 2011; Omara et al. 2012; Rocha et al. 2013). Interestingly, treatment with WSMoL resulted in reduced liver and kidney damage, as reflected by blood biochemical parameters and histological analysis, likely through reversal of the sustained hyperglycemic state.

Lectins, including those derived from plants, have been reported to exert antihyperglycemic effects by acting directly on pancreatic β‐cells, modulating the expression of genes involved in glucose metabolism, interacting directly with insulin receptors, enhancing peripheral glucose uptake, and mimicking insulin action in target tissues (Cuatrecasas and Tell 1973; Khalid et al. 1989; Bajaj et al. 2011; Sawant et al. 2017; Paula, Sousa, et al. 2017; Paula, Oliveira, et al. 2017; Alves et al. 2020; Simha et al. 2024). Paula, Sousa, et al. (2017) and Paula, Oliveira, et al. (2017) reported that plant proteins in *M. oleifera* leaves and seeds exhibit insulin‐like activity, capable of interacting with insulin receptors and modulating glycemic signaling pathways. WSMoL, when administered by gavage (5 mg/kg) for 21 consecutive days to C57BL/6 mice induced to develop T2DM through a combination of a high‐fat diet and streptozotocin, reduced glycemic levels to values comparable to those of nondiabetic mice and decreased insulin resistance (Vera‐Nunez et al. 2021). Although the exact mechanism of WSMoL in the alloxan model remains unclear, this evidences from the literature points to possible roles in stimulating insulin secretion, protecting pancreatic β‐cells, or exerting insulin‐mimetic activity, and can guide future studies.

Collectively, the data indicate that WSMoL administration in hyperglycemic animals led to improvements in liver and kidney histological damage associated with the T1DM‐like condition. In contrast, its administration at all tested doses—including those considered therapeutic—to healthy animals was linked to evidence of persistent inflammatory changes in these organs. These findings suggest that the physiological context can modulate the effects of WSMoL and that its therapeutic benefits could potentially outweigh the observed side effects. Nevertheless, further studies are warranted to investigate this possibility, particularly through experiments involving prolonged administration periods and assessments of potential adaptive or transient responses.

## 5 Conclusion

Administration of WSMoL at doses of 10, 25, and 50 mg/kg, whether as a single or repeated intraperitoneal treatment, demonstrated a favorable safety profile, as evidenced by the absence of mortality, behavioral changes, or major alterations in food intake, body weight, and most hematological and biochemical parameters. However, the mild inflammatory responses observed in the liver, kidney, and spleen indicate that low‐level tissue stress may occur and warrant careful monitoring during prolonged use. In the T1DM‐like model, WSMoL produced significant hypoglycemic effects, improving fasting blood glucose and glucose tolerance, and ameliorating diabetes‐associated hepatic and renal alterations. Together, these findings suggest that WSMoL is a promising natural candidate for the management of diabetes, although potential subclinical toxicity should be considered. These results stimulate further studies to delineate its mechanisms of action—including effects on insulin secretion, insulin signaling pathways, glucose transporter expression, and oxidative stress modulation—and to evaluate its long‐term safety profile.

## Author Contributions

**Alícia Natalie Silva dos Santos:** conceptualization, methodology, formal analysis, investigation, writing – original draft, and visualization. **Matheus Cavalcanti de Barros:** methodology, formal analysis, investigation, and visualization. **Cleidiane Silva Vieira de Souza:** investigation. **Ryan Cristian da Silva:** formal analysis, investigation, and visualization. **Luana Cassandra Breitenbach Barroso Coelho:** resources. **Maria Tereza dos Santos Correia:** resources. **Fernanda das Chagas Ângelo Mendes Tenório:** methodology, validation, formal analysis, resources, writing – review and editing, visualization, and funding acquisition. **Alisson Macário de Oliveira:** data curation. **Thiago Henrique Napoleão:** validation, formal analysis, resources, writing – review and editing, visualization, project administration, and funding acquisition. **Robson Raion de Vasconcelos Alves:** conceptualization, methodology, validation, formal analysis, investigation, data curation, writing – original draft preparation, visualization, and supervision. **Patrícia Maria Guedes Paiva:** conceptualization, methodology, validation, formal analysis, resources, writing – review and editing, visualization, supervision, project administration, and funding acquisition.

## Funding

This work was supported by the Fundação de Amparo à Ciência e Tecnologia do Estado de Pernambuco (APQ‐0108‐2.08/14, APQ‐2144‐2.08/24), the Conselho Nacional de Desenvolvimento Científico e Tecnológico (407192/2018‐2), and the Coordinação de Aperfeiçoamento de Pessoal de Nível Superior (Finance Code 001).

## Conflicts of Interest

The authors declare no conflicts of interest.

## Data Availability

The data that support the findings of this study are available from the corresponding author upon reasonable request.
